# Training to identify red flags in the acute care of trauma: who are the patients at risk for early death despite a relatively good prognosis? An analysis from the TraumaRegister DGU®

**DOI:** 10.1186/s13017-020-00325-0

**Published:** 2020-08-03

**Authors:** Philip-C. Nolte, David Häske, Rolf Lefering, Michael Bernhard, Sebastian Casu, Susanne Frankenhauser, Andreas Gather, Paul A. Grützner, Matthias Münzberg, the TraumaRegister DGU

**Affiliations:** 1 Department of Trauma and Orthopedic Surgery, BG Trauma Center Ludwigshafen, 67071 Ludwigshafen, Germany; 2 Center for Public Health and Health Services Research, University Hospital Tübingen, 72076 Tübingen, Germany; 3 German Red Cross, Emergency Medical Service, 72764 Reutlingen, Germany; 4 Institute for Research in Operative Medicine (IFOM), University Witten/Herdecke, Cologne, Germany; 5 Emergency Department, University Hospital Duesseldorf, 40225 Duesseldorf, Germany; 6 Department of Intensive Care and Emergency Medicine, Helios Hospital Salzgitter, 38226 Salzgitter, Germany; 7 Department of Rescue and Emergency Medicine, BG Trauma Center Ludwigshafen, 67071 Ludwigshafen, Germany; 8 Committee on Emergency Medicine, Intensive Care and Trauma Management (Sektion NIS) of the German Trauma Society (DGU), Berlin, Germany

**Keywords:** Trauma, Revised injury severity classification (RISC-score), Trauma registry, Early death, Red flags, Prehospital, Life support

## Abstract

**Background:**

In the acute care of trauma, some patients with a low estimated risk of death die suddenly and unexpectedly. In this study, we aim to identify predictors for early death within 24 h following hospital admission in low-risk patients.

**Methods:**

The TraumaRegister DGU® was used to collect records of patients who were primarily treated in a participating hospital between 2004 and 2013 with a RISC II score below 10%.

**Results:**

During the study period, 64,379 patients met the inclusion criteria. The mean RISC II score was 2.0%, and the mean ISS was 16 ± 9. The overall hospital mortality rate was 2.1%, and 0.5% of patients (*n* = 301) died within the first 24 h. A SPB of ≤ 90 mmHg was associated with an increased risk of death (*p* < 0.001). An AIS abdomen score of ≥ 3 was associated with increased risk of death within the first 24 h (*p* < 0.001). A high risk of early death was also seen in patients with an AIS score (thorax) ≥ 3; 51% of those who died died within the first 24 h (*p* < 0.005). Death in patients over 60 years was more common after 24 h (*p* < 0.001). Patients with an ASA score of ≥ 3 were more likely to die after the first 24 h (*p* < 0.001).

**Conclusions:**

Indicators predicting a high risk of early death in patients with a low RISC II score include a SPB ≤ 90 mmHg and severe chest and abdominal trauma. Emergency teams involved in the acute care of trauma patients should be aware of these “red flags” and treat their patients accordingly.

## Introduction

Injuries account for more than five million annual deaths worldwide [1]. The impact on the younger population is more severe, where trauma is the leading cause of death among those aged 15–29 years [1]. Patients with extensive injuries and, therefore, a high Injury Severity Score (ISS) have decreased chances of survival [2]. It is broadly accepted that these severely injured patients need to be treated with great alertness and awareness of their fragile situation, receiving extensive therapy.

For prognostication and the adjustment of mortality rates, not only the anatomical injuries as displayed by the ISS are important, but also the physiological responses to injury. Thus, the Revised Injury Severity Classification (RISC) score was developed and updated to its 2nd version (RISC II) in 2014 [3, 4]. The RISC II score was shown to be superior to the existing scoring systems and is used in the context of the TraumaRegister DGU® (TR-DGU) for outcome adjustment [4]. Patients presenting with low RISC II scores have higher survival chances, but experience shows that there are patients who seem to fall through the cracks and, despite good prognosis and seemingly no disastrous injuries, die suddenly and unexpectedly [5–7]. If those cases ultimately die, and especially if they die shortly after hospital admission, a detailed analysis might help to understand the life-threatening mechanisms in trauma. Therefore, this study aimed to identify “red flags” (indicators signaling danger) in the acute care of trauma patients that account for a high risk of early death, despite their good prognosis.

## Materials and methods

### Database

The TraumaRegister DGU® (TR-DGU) of the German Trauma Society (Deutsche Gesellschaft für Unfallchirurgie, DGU) was founded in 1993. The aim of this multi-center database is to collect pseudonymized and standardized documentation of severely injured patients.

Data are collected prospectively in four consecutive time phases from the site of the accident until discharge from hospital: (A) prehospital phase, (B) emergency room and initial surgery, (C) intensive care unit, and (D) discharge. The documentation includes detailed information on demographics, injury pattern, comorbidities, pre- and in-hospital management, a course on the intensive care unit, and relevant laboratory findings (including data on transfusion and case outcomes). The inclusion criterion is admission to hospital via an emergency room with subsequent ICU/IMC care or reaching the hospital with vital signs and die before admission to the ICU.

The infrastructure for documentation, data management, and data analysis is provided by the AUC—Academy for Trauma Surgery (AUC—Akademie der Unfallchirurgie GmbH), a company affiliated to the German Trauma Society. The scientific leadership is provided by the Committee on Emergency Medicine, Intensive Care and Trauma Management (Sektion Notfall-, Intensivmedizin und Schwerverletztenversorgung (NIS)) of the German Trauma Society. The participating hospitals submit their pseudonymized data into a central database via a web-based application. Scientific data analysis is approved according to a peer review procedure established by Sektion NIS.

The participating hospitals are primarily located in Germany (90%), but an increasing number of hospitals in other countries also contribute data (including Austria, Belgium, China, Finland, Luxembourg, Slovenia, Switzerland, the Netherlands, and the United Arab Emirates). Currently, approximately 35,000 cases from nearly 700 hospitals are entered into the database each year. Participation in the TraumaRegister DGU® is voluntary. The TraumaNetwork DGU® is a network of hospitals which are organized according to uniform standards of care and quality assurance and are according to the DGU® white book of trauma care (Weißbuch Schwerverletztenversorgung) based on their structure, resources in staff, equipment, and tasks, resulting in level 1 to 3 trauma centers [8]. Level 1 trauma centers represent centers with the highest resources, whereas level 3 centers have limited resources. For hospitals associated with TraumaNetwork DGU®, however, the entry of at least a basic data set is obligatory for reasons of quality assurance. The present study follows the publication guidelines of the TR-DGU and is registered as TR-DGU project TR-DGU-2014-059.

### Prognostic score

The Revised Injury Severity Classification (RISC) score was developed and used for outcome adjustment in the context of the TraumaRegister DGU® since 2003 [3].

The currently used RISC II (Revised Injury Severity Classification, version 2) score was developed in more than 30,000 cases from the TR-DGU documented in 2010 and 2011, and repeatedly validated in the following years. It consists of the following predictors: AIS (Abbreviated Injury Scale) severity level of worst and second-worst injury, head injury, age, sex, pupil reactivity and size, pre-injury health status, trauma mechanism (penetrating), blood pressure, acidosis (base deficit), coagulopathy (INR), hemoglobin, and cardiopulmonary resuscitation [4]. Missing values are included as a separate category for all variables except for age and injuries. The RISC II score was superior to the existing scoring systems, including the original RISC score [4].

### Patient selection

Primary admitted patients treated in a participating German hospital between 2004 and 2013 were included if their RISC II prognosis was below 10% risk of death. Further exclusions were age = 0, early transfer out within 48 h (no outcome available), ISS < 4 (RISC not validated), and minor injuries (maximum AIS ≤ 2) not treated on intensive care unit (ICU). Patients were divided into survivors and non-survivors, and non-survivors were further divided into early deaths (< 24 h) and late deaths.

### Outcome indicators

Various common variables were tested for their ability to serve as early indicators (“red flags”) of death. The variables were grouped into the subcategories “patient-related indicators” [American Society of Anesthesiologists (ASA) score and age], vital sign-related indicators [first prehospital Glasgow Coma Scale (GCS), pre- and intrahospital systolic blood pressure (SBP)], system-related indicators (air rescue vs. ground rescue, admission during on-call hours), level of trauma center (level 1 to 3), and injury-related indicators (AIS head ≥ 3, AIS thorax ≥ 3, AIS abdomen ≥ 3, AIS pelvis ≥ 4, AIS extremities ≥ 3).

### Statistical analysis

Data are presented as mean with standard deviation (± SD) and median for continuous variables and as percentages for categorical variables. Pairwise comparisons (survivor vs. non-survivor, and early vs. late deaths) were performed with the chi-squared test and Mann-Whitney *U* test, as appropriate. Statistical analysis was performed using SPSS, version 24.0 (IBM Inc., Armonk, NY, USA). *p* values ≤ 0.05 were considered statistically significant.

## Results

A total of 64,379 patients (72.7% male, mean age 44 years) met the criteria and were included. The hospital mortality rate was 2.1% (*n* = 1,372), and 301 of these (0.5% of all cases or 22% of those who died) died within the first 24 h of hospital admission.

The mean RISC II score for all included patients was 2.0% and matched well with the observed mortality rate. The average ISS was 16.4 ± 9.2, and 49% fulfilled the ISS ≥ 16 criterion. The RISC II score and the ISS were highest (5.9%; 22.0 ± 12.3) in the group of patients who died within 24 h. The type and mechanism of trauma were typical for a western-European country with mainly blunt trauma (95.7%), and with over half of them (60.1%) being traffic related. The basic demographic data and parameters are presented in Table 1.

**Table 1:** Survivorship according to demographic data and outcome indicators

		Survivor	Non-survivor	***p*** value	Early deaths < 24 h	Late deaths > 24 h	***p*** value
**Age ≥ 60 years**	*n* (%)	13,887 (22.0)	749 (54.6)	***p*< 0.001**	126 (41.9)	623 (58.2)	***p*< 0.001**
**ASA 3–4**	*n* (%)	3987 (8.7)	346 (35.9)	***p*< 0.001**	47 (23.4)	299 (39.2)	***p*< 0.001**
**ISS**	Mean ± SD	16.3 ± 9.1	20.7 ± 11.0	***p*< 0.001**	22.0 ± 12.3	20.3 ± 10.5	***p*= 0.083**
**RISC II** predicted mortality	Mean ± SD	1.9 ± 2.3	5.3 ± 2.9	***p*< 0.001**	5.9 ± 2.8	5.1 ± 2.9	***p*< 0.001**
**Air rescue**	*n* (%)	15,527 (25.4)	341 (25.6)	***p*= 0.912**	68 (23.4)	273 (26.2)	***p*= 0.328**
**Admission during on-call hours**	n (%)	42,108 (67.6)	854 (63.6)	***p*= 0.002**	201 (68.4)	653 (62.2)	***p*= 0.054**
**Trauma center**							
**Level 1**	*n* (%)	38,518 (61.1)	878 (64.0)	***p*= 0.08**	165 (54.8)	713 (66.6)	***p*< 0.001**
**Level 2**		20,085 (31.9)	411 (30.0)		109 (36.2)	302 (28.2)	
**Level 3**		4404 (7.0)	83 (6.0)		27 (9.0)	56 (5.2)	
**SBP ≤ 90 mmHg, prehospital**	*n* (%)	5021 (8.9)	175 (14.5)	***p*< 0.001**	67 (25.9)	108 (11.4)	***p*< 0.001**
**SBP ≤ 90 mmHg, on admission**	*n* (%)	2736 (4.8)	125 (10.3)	***p*< 0.001**	54 (21.3)	71 (7.4)	***p*< 0.001**
**GCS ≤ 8, prehospital**	*n* (%)	5657 (9.5)	261 (20.3)	***p*< 0.001**	74 (26.5)	187 (18.6)	***p*= 0.004**

*ASA* American Society of Anesthesiologists, *BE* base excess, *GCS* Glasgow Coma Scale, *Hb* hemoglobin, *ISS* Injury Severity Score, *RISC II* Revised Injury Severity Classification version 2, *SBP* systolic blood pressure

### Patient-related indicators

In the non-survivor group, patients with an age of 60 years and above died significantly more often after 24 h [58.2% (*n* = 623) vs. 41.9% (*n* = 126) within 24 h; *p* < 0.001]. Based on the ASA classification, 4333 (9.3%) patients had an ASA of ≥ 3 before the accident. The evaluation showed that patients with an ASA classification ≥ 3 were more likely to die after 24 h than within the first 24 h (*p* < 0.001).

### Vital sign-related indicators

A prehospital GCS of 8 or below was documented in 5918 cases (9.8%). Of the 261 patients with GCS ≤ 8 who died in hospital, only 74 died within 24 h (*p* = 0.004).

The mortality of patients with a prehospital SBP ≤ 90 mmHg was 14.5% (*n* = 175), which was significantly higher compared to shocked patients in survivors (8.9%; *n* = 5021; *p* < 0.001). In patients who died within 24 h, a prehospital SBP ≤ 90 mmHg was documented in 25.9% and, therefore, significantly more often than in patients who died later (11.4%, *p* < 0.001).

A total of 10.3% (*n* = 125) of the patients who died in the hospital, but only 4.8% (*n* = 2736) of survivors had an intrahospital SBP ≤ 90 mmHg (*p* < 0.001). In patients who died within 24 h, an intrahospital SBP ≤ 90 mmHg was documented in 21.3%, which is significantly more frequent than in those who died later (7.4%, *p* < 0.001).

### System-related indicators

There was no difference in mortality when comparing air rescue vs. ground transportation (*p* = 0.912). Patients who died within 24 h were similarly distributed between the groups (ground rescue 0.5% vs. air rescue 0.4%; *p* = 0.328).

There were a total of 42,962 (67.5%) patients admitted to the hospital during on-call hours, of which 854 (2.0%) died. During regular hours, 489 of 20,653 (2.4%) patients died, which was significantly more than within on-call hours (*p* = 0.002). Of all patients who died during on-call hours, 23.5% died within the first 24 h, whereas during regular work hours only 19.0% of patients died within 24 h (*p* = 0.054).

Most patients [39,396 (61.2%)] were admitted to a level 1 trauma center, 20,496 (31.8%) to a level 2 center, and 4487 (7.0%) to a level 3 center (*p* = 0.08). Of all patients who died within the first 24 h, mortality was higher in the level 3 trauma centers (32.5%) compared to the level 1 (18.7%) and 2 (26.5%) trauma centers (*p* < 0.001).

### Injury-related indicators

Details to the injury patterns are displayed in Table 2. A total of 16,730 (26.0%) patients had a relevant head injury (AIS ≥ 3). Patients who died had significantly more head injuries (39.4%) than survivors (25.7%, *p* < 0.001). However, among the 541 patients who died with an AIS head ≥ 3, there was no significant difference between early and late deaths (*p* = 0.196).

**Table 2:** Survivorship according to injury-related indicators

Relevant injury (AIS ≥ 3)		Survivor	Non-survivor	***p*** value	Early deaths < 24 h	Late deaths > 24 h	***p*** value
**Head**	*n* (%)	16,189 (25.7)	541 (39.4)	***p*< 0.001**	109 (36.2)	432 (40.3)	***p*= 0.196**
**Thorax**	*n* (%)	24,345 (38.6)	635 (46.3)	***p*< 0.001**	154 (51.2)	481 (44.9)	***p*< 0.005**
**Abdomen**	*n* (%)	6775 (10.8)	227 (16.5)	***p*< 0.001**	75 (24.9)	152 (14.2)	***p*< 0.001**
**Pelvis (AIS ≥ 4)**	*n* (%)	3315 (5.3)	115 (8.4)	***p*< 0.001**	32 (10.6)	83 (7.7)	***p*= 0.111**
**Extremities**	*n* (%)	17,155 (27.2)	429 (31.3)	***p*< 0.001**	95 (31.6)	334 (31.2)	***p*= 0.901**

*AIS* Abbreviated Injury Scale

A total of 24,980 (38.8%) patients had an AIS thorax ≥ 3. In the non-survivor group, 46.3% of patients had an AIS thorax ≥ 3, compared to 38.6% in the survivor group (*p* < 0.001). Patients with an AIS thorax ≥ 3 died significantly quicker than those with a lower AIS thorax score (*p* < 0.005).

There were 7002 (10.9%) patients with an AIS abdomen ≥ 3, of which significantly more died than survived (*p* < 0.001). Also, patients with severe abdominal injuries died quicker (24.9% within 24 h vs. 14.2% after 24 h; *p* < 0.001).

The mortality rate was higher among the 3430 patients with an AIS pelvis ≥ 4. Here, of the non-survivors, 115 (8.4%) had an AIS pelvis of ≥ 4, while among the survivors only 5.3% had an AIS ≥ 4 (*p* < 0.001). However, there was no significant difference between death before or after 24 h (*p* < 0.111).

Among all patients, 27.3% (*n* = 17,584) had an AIS extremities score ≥ 3. Also, in patients with severely injured extremities, death was significantly more frequent than survival (31.3% vs. 27.2%; *p* < 0.001), but no difference was detected in the temporal distribution.

## Discussion

The death of a patient with a good prognosis was often described as an “unexpected” death. However, a prognosis is nothing more than an estimated probability, that is, if one out of 10 patients each with a prognosis of 10% dies, then this is exactly what the score expected. However, in individual cases that died, it would be very important to understand why these patients died, despite the absence of critical findings. This study aimed to identify predictors for early death in low-risk patients. We found that a systolic blood pressure ≤ 90 mmHg in the pre- or intrahospital setting, as well as severe chest and abdominal trauma, as represented by an AIS ≥ 3, could account for a high risk of early death in patients with a RISC II score below 10%. Those indicators should especially alert emergency teams in the trauma room and can be recognized as “red flags” in the acute care of trauma patients.

Higher age is an independent risk factor for trauma-related death; patients are twice as likely to die if they are 65 years and older [7–11]. In older patients, the preexistence of typical age-dependent medical conditions, such as diabetes, high blood pressure, reduced respiratory, and cardiac capacity, as well as polypharmacy, is the rule rather than the exception [12]. Following trauma, those conditions contribute to an increased risk of complications, such as sepsis and multi-organ failure [9, 12], thereby contributing to a higher mortality rate [13]. In line with the literature, we detected significantly higher mortality in the 60-year-old or above patient group. However, whereas overall mortality was higher in the patients aged 60 years and above, younger patients were more prevalent in the early death group. We suggest that this phenomenon is most likely due to the different mechanisms of injury (e.g., high-velocity motor vehicle accident) in the younger patients, followed by higher injury severities. Also, younger trauma victims might better compensate for the consequences of an injury that makes them look quite normal on admission (i.e., the values used for prediction). The higher overall mortality in the elderly might also not derive from the trauma itself but from independent medical conditions or late complications, such as sepsis or multi-organ failure. The coexistence of higher age and medical conditions also explains our findings regarding the ASA score. Our results show that patients with an ASA score ≥ 3 were more likely to die. Furthermore, similar to the higher age, patients with an ASA score ≥ 3 died significantly more often after 24 h than in the first 24 h following hospital admission. In older patients with low RISC II scores, care should be taken to avoid late complications occurring after 24 h, such as sepsis, while younger patients are at risk of sudden death, most likely due to higher injury severity. However, we did not find a higher age or ASA score to be a “red flag” for death within 24 h.

Various studies have shown that traumatic brain injury is one of the leading causes of death due to trauma and was found to be responsible for about half of them [6, 14, 15]. In the non-survivor group of our study collective, there were significantly more patients with a prehospital GCS ≤ 8. However, patients with a GCS ≤ 8 were more likely to die after the first 24 h; therefore, a GCS ≤ 8 was not considered to be a “red flag” for early death. These findings are concordant with those of prior studies, demonstrating that death due to traumatic brain injury is not the predominant cause in the early hours following trauma [6, 7, 14].

Importantly, we were able to show that hypotensive shock, as represented by a prehospital and intrahospital SBP ≤ 90 mmHg, accounts for significantly higher risk of death in general and within 24 h in particular. We, therefore, consider a low SBP to be an important “red flag.” Since hypotensive shock in trauma patients is most likely due to hemorrhage, this becomes especially relevant because Kleber et al. found that death by exsanguination is preventable in 73.1% of patients [16].

There is ongoing discussion about whether air rescue or ground rescue is more beneficial for trauma patients. Recent literature shows increasing evidence that air rescue might be superior to ground rescue in major trauma [16–18]. However, a review from 2015 could not determine an accurate benefit for air rescue [17]. In our collection of low RISC II patients, we could not detect a difference in survival rate when comparing air and ground rescue; both subgroups had a mortality rate of 2.1%. Also, no difference was detected in the temporal distribution of death.

To evaluate whether there is a so-called weekend effect, we compared the mortality of patients who were admitted to the hospital during on-call hours to those who were admitted during regular work hours. Mortality was significantly lower following admission during on-call hours. There was no significant difference when comparing early vs. late deaths, although a tendency towards higher mortality within 24 h was seen in patients admitted during on-call hours. These results are in line with the findings of other groups who found no difference or even lower mortality during weekends/weeknights (on-call hours) compared to weekdays [18–20]**.** A possible explanation for this finding could be that during on-call hours, a higher awareness for critically injured patients and less distraction from routine daytime work exist.

We could not detect a difference in mortality between the three types of trauma centers, even though patients admitted to a level 1 trauma center had better chances to survive the first 24 h compared to level 2 or 3 trauma centers; however, no significant differences were found in overall mortality. This effect might be due to more aggressive treatment in a level 1 center, which allows patients to survive the first 24 h.

Severe chest and abdominal trauma have been described to account for high mortality within 1 and 6 h, respectively, in a trauma center with over one third of patients presenting with penetrating trauma [21]. Interestingly, in a trauma center in the Netherlands with more than 90% blunt trauma, chest injury was also one of the major causes of early death [6]. Our results are in line with these findings, as we found a severe chest and abdominal injury, as represented by an AIS ≥ 3, to be responsible for high mortality and early deaths. Given the observation, that death following abdominal trauma is most likely due to hemorrhage [22]. Our finding stresses the importance of thorough initial examination and continuous reevaluation (e.g., clinical and sonography) of patients with abdominal trauma. These patients may present with sufficient vital parameters on admission but become worse over a short period of time. Likewise, blunt chest trauma is challenging to treat as it can cause a wide variety of injuries (e.g., pneumothorax, pericardial effusion, aortic injuries, myocardial contusion) and symptoms, and is referred to as “clinical chameleon” [23]. Again, patients can present hemodynamically stable on initial evaluation although suffering a life-threatening injury. Therefore, care should be taken not to underestimate the severity of the injury.

An AIS ≥ 3 of the head and the extremities and an AIS ≥ 4 of the pelvis were all associated with significantly higher mortality, though no statistical difference was found between early or late deaths, and therefore, those parameters did not qualify as “red flags” in the acute care of trauma patients.

Similar impressions as our results were described in the literature, but not with a focus on patients with a statistically good prognosis [7]. In addition to the statistical results, however, it is at least as important how these findings can be integrated into the training and clinical knowledge of health care professionals. It is well known that knowledge flattens with time and can only be maintained through lifetime learning [23–25]. It is also in the hands of those responsible in the emergency medical services, shock room, and hospital to pass on this knowledge. The results of our study make it visible how crucial clinical reasoning is, especially for young professionals, on their way to becoming experts [26]. One must not be blinded by initially good vital signs, but must always see clinical impression, vital values, and medical knowledge in combination.

### Limitations

Our study has limitations. The data were collected from the TraumaRegister DGU® retrospectively and are therefore considered to be less valid than data from prospective trials. Also, unlike in most clinical trials, testing for data correctness in the TR-DGU is only performed in a small sample of cases. Furthermore, assessed variables such as prehospital or intrahospital blood pressure may have been subject to different prehospital procedures (infusion management, blood components) that vary between countries.

## Conclusion

Based on our analyses, indicators predicting a high risk of early death within 24 h in patients with a low RISC II score are a systolic blood pressure ≤ 90 mmHg in the prehospital or intrahospital setting, as well as severe chest and abdominal trauma, as represented by an AIS ≥ 3. Emergency teams involved in the acute care of trauma patients should be aware of those “red flags” and treat their patients accordingly.

## Data Availability

None

## Abbreviations

AIS: Abbreviated Injury Scale
ASA: American Society of Anesthesiologists
AUC: Akademie der Unfallchirurgie GmbH/Academy for Trauma Surgery
DGU: Deutsche Gesellschaft für Unfallchirurgie/ German Trauma Society
ICU: Intensive care unit
IMC: Intermediate care unit
ISS: Injury Severity Score
RISC II: Revised Injury Severity Classification, version 2
SBP: Systolic blood pressure
TR-DGU: TraumaRegister DGU®
Sektion NIS: Committee on Emergency Medicine, Intensive Care and Trauma Management of the German Trauma Society
